# Complement factor C5a induces atherosclerotic plaque disruptions

**DOI:** 10.1111/jcmm.12357

**Published:** 2014-08-15

**Authors:** Anouk Wezel, Margreet R de Vries, H Maxime Lagraauw, Amanda C Foks, Johan Kuiper, Paul HA Quax, Ilze Bot

**Affiliations:** a Division of Biopharmaceutics, Leiden Academic Centre for Drug Research, Leiden UniversityLeiden, The Netherlands; b Department of Surgery, Leiden University Medical CenterLeiden, The Netherlands; c Einthoven Laboratory for Experimental Vascular Medicine, Leiden University Medical CenterLeiden, The Netherlands

**Keywords:** C5a, atherosclerosis, apoptosis, plaque rupture

## Abstract

Complement factor C5a and its receptor C5aR are expressed in vulnerable atherosclerotic plaques; however, a causal relation between C5a and plaque rupture has not been established yet. Accelerated atherosclerosis was induced by placing vein grafts in male apoE^−/−^ mice. After 24 days, when advanced plaques had developed, C5a or PBS was applied locally at the lesion site in a pluronic gel. Three days later mice were killed to examine the acute effect of C5a on late stage atherosclerosis. A significant increase in C5aR in the plaque was detectable in mice treated with C5a. Lesion size and plaque morphology did not differ between treatment groups, but interestingly, local treatment with C5a resulted in a striking increase in the amount of plaque disruptions with concomitant intraplaque haemorrhage. To identify the potential underlying mechanisms, smooth muscle cells and endothelial cells were treated *in vitro* with C5a. Both cell types revealed a marked increase in apoptosis after stimulation with C5a, which may contribute to lesion instability *in vivo*. Indeed, apoptosis within the plaque was seen to be significantly increased after C5a treatment. We here demonstrate a causal role for C5a in atherosclerotic plaque disruptions, probably by inducing apoptosis. Therefore, intervention in complement factor C5a signalling may be a promising target in the prevention of acute atherosclerotic complications.

## Introduction

Rupture of an atherosclerotic plaque is the main cause of myocardial infarction or stroke, which are still the leading causes of death worldwide 1,2. Features of an unstable plaque, prone to rupture, are a large lipid core covered by a thin fibrous cap depleted of smooth muscle cells. The exact mechanism of progression from an asymptomatic stable lesion to a vulnerable plaque with subsequent rupture is still incompletely understood, but apoptosis of smooth muscle cells and endothelial cells is thought to be crucial 3–5. In search for new therapeutic strategies to reduce the incidence of plaque rupture, attention turned to the complement system, in particular to complement factor C5a.

The complement system, consisting of ∼30 proteins, has been suggested to play an important role in cardiovascular diseases, among which atherosclerosis 6,7. The anaphylatoxin C5a, a split product of C5, is constantly produced during complement activation. C5a is one of the most potent inflammatory chemoattractants and promotes leucocyte recruitment to sites of inflammation 8–11. Furthermore, C5a has the ability to activate endothelium and induce the expression and release of numerous cytokines and chemokines such as CCL2 12,13. Various cell types, such as macrophages, endothelial cells, smooth muscle cells and mast cells, express receptors for C5a. Previously, we have shown that vein graft disease is aggravated by C5a as a result from attracting and activating mast cells 14. Others have demonstrated that inhibition of C5aR signalling reduces atherosclerosis and neointima formation in apoE^−/−^ mice 15,16. As a result of its pronounced effects on a number of cell types within the atherosclerotic plaque, C5a may also be one of the key components in plaque destabilization and acute plaque rupture. Previously, it has been demonstrated that elevated levels of C5a are associated with increased cardiovascular risk in patients with advanced atherosclerosis 17. However, since that study was mere observational, a direct causal role for C5a could not be assigned.

A major drawback in pre-clinical research aiming at the identification of factors involved in acute cardiovascular syndromes is the lack of suitable atherosclerotic mouse models that resemble the complex human atherosclerotic plaque morphology with concomitant plaque rupture. Interestingly, we have recently established that lesions induced in a murine vein graft model are more complex and display both plaque disruptions and intraplaque haemorrhages 18, which are important features of acute atherosclerotic plaque destabilization in humans. These findings render this an excellent model to study late stage atherosclerosis and acute complications.

In this study, we thus aimed to investigate the acute effect of complement factor C5a on late stage atherosclerosis and concomitant plaque complications in this mouse model.

## Material and methods

### Mice

This study was performed in compliance with Dutch government guidelines and the Directive 2010/63/EU of the European Parliament. All animal experiments were approved by the animal welfare committee of the Leiden University Medical Center (approval reference number 10091). Male apoE^−/−^ mice (PBS/PBS treated mice: *n* = 8; C5a/PBS treated mice: *n* = 9; C5a/cromolyn treated mice: *n* = 7), were obtained from the Gorlaeus Laboratory animal breeding facility (Leiden) and were given water and chow *ad libitum*. Plasma total cholesterol levels were measured by enzymatic procedures using precipath standardized serum as an internal standard (Boehringer, Mannheim, Germany).

### Surgical intervention

Before surgery, gel placement and sacrifice, mice were anaesthetized by an intra-peritoneal injection with midazolam (5 mg/kg; Roche, Woerden, The Netherlands), domitor (0.5 mg/kg; AST Farma, Oudewater, The Netherlands) and fentanyl (0.05 mg/kg; Janssen, Beerse, Belgium). After surgery, mice were antagonized with a subcutaneous injection of flumazenil (0.5 mg/kg; Fresenius Kabi, Schelle, Belgium), antesedan (2.5 mg/kg; AST Farma) and buprenorphine (0.1 mg/kg; MSD Animal Health, Boxmeer, The Netherlands). Vein graft surgery was performed to induce advanced atherosclerotic lesions. In brief, the carotid artery of the recipient mice was cut in the middle; both ends of the artery were everted around cuffs and ligated with 8.0 sutures. The vena cava from donor littermates was harvested and sleeved over the cuffs. By placing the vein in the arterial circulation with high blood pressure, endothelial damage was induced with subsequent accelerated atherosclerosis. After 24 days when advanced lesions were present, age and weight-matched mice were anaesthetized. A F-127 pluronic gel (25% w/v) was placed in the surrounding tissue of the vein graft (the perivascular tissue) completely surrounding the vessel, enabling us to investigate the local effect rather than the systemic effect of our compound. The gel contained either C5a (5 μg; Hycult Biotech, Uden, The Netherlands) as has been previously described 14, or PBS.

As we have previously established that C5a may exert its effects on atherosclerosis *via* activation of mast cells 14, we have treated a subgroup of C5a challenged mice with the mast cell stabilizer cromolyn to identify mast cell dependent effects. Thus, from gel placement until sacrifice at day 28, mice received daily intraperitoneal injections with a commonly used mast cell stabilizer cromolyn (50 mg/kg/day; Sigma-Aldrich, Zwijndrecht, The Netherlands) 19,20 or PBS.

### Histological and immunohistochemical analysis

Cross-sections of paraffin embedded vein grafts (5 μm thick) were stained with haematoxylin-phloxine saffron for measurement of lesion size, fibrin content and plaque dissection analysis. Fibrin content was graded by two blinded independent investigators on a scale from 0 to 3, with 0 representing no fibrin and 3 representing severe transmural fibrin depositions. Collagen content was visualized with a picrosirius red staining. Mast cell and neutrophil staining was performed with an enzymatic chloroacetate esterase kit (Sigma-Aldrich); when granules were apparent in the vicinity of the mast cell they were scored as activated. Neutrophils were stained light pink, while the segmented nuclei were visualized by Gill's haematoxilin 21. Composition of the atherosclerotic lesion was further evaluated by immunohistochemical stainings for macrophages (MAC3, 1:200; BD-Pharmingen, San Diego, CA, USA), smooth muscle cell actin (1:1000; Sigma-Aldrich); C5a (1:400; Hycult Biotechnology) and C5aR (1:400; SantaCruz, Dallas, Texas, USA). A TUNEL staining was performed according to manufacturer's protocol to detect apoptotic cells in the atherosclerotic plaque (*in situ* cell death detection kit, POD, Roche). The total amount of cells in the vessel wall area, as well as the amount of apoptotic cells, was counted manually, after which the percentage of apoptosis was calculated. All staining measurements were performed on six consecutive cross-sections of the vein grafts, ∼150 μm spaced, in a blinded manner by a single observer.

Plaque dissection analysis was determined over a total vein graft length of 1800 μm. The disruptions were defined as a connection or fissure between the lumen and part of the vessel wall underneath the adventitia, filled with fibrin and erythrocytes. Quantification of the lesion area and immunostained positive area were performed with computer assisted software (Qwin; Leica, Cambridge, UK). In brief, the total intimal area was measured, as well as the stained area. The stained area was than calculated as a percentage of the total intimal area.

### Cell culture

To generate bone marrow derived macrophages (BMDMs), cells were isolated from bone marrow of C57Bl/6 mice and cultured for 7 days in RPMI medium supplemented with 20% foetal calf serum (FCS), 2 mmol/l l-glutamine, 100 U/ml penicillin, 100 μg/ml streptomycin (all from PAA, Colbe, Germany) and 30% L929 cell-conditioned medium [as the source of macrophage colony-stimulating factor (M-CSF)] as has been described previously 22–24. Primary cultured murine smooth muscle cells (vSMC) 25 and a murine cell line for endothelial cells H5V 26 were cultured in DMEM medium supplemented with 10% FCS, 2 mmol/l l-glutamine, 100 U/ml penicillin and 100 μg/ml streptomycin.

### Collagen synthesis assay

To measure collagen production by vSMC, cells were seeded at a density of 0.2 × 10^6^ cells per well. Control medium or medium containing 0.2, 2 or 20 nM C5a was added after attachment of the cells. Also, 1 μCi [^3^H]Proline (Perkin Elmer, Groningen, The Netherlands) together with of 50 μg/ml ascorbic acid was added and incubated overnight at 37°C. Cells were taken up in 20 mM Tris HCl/0.36 mM CaCl_2_ (pH = 7.6) and sonicated for 2 min. Collagen was degraded by incubation with 100 U/ml collagenase for 2 hrs at 37°C, after which samples were centrifuged for 15 min. at maximum speed. Proteins were precipitated for 30 min. on ice using 50% trichloroacetic acid, after which [^3^H]Proline content in the supernatant as a measure for collagen production was quantified in a liquid scintillation analyzer (Packard 1500 Tricarb, Downers Grove, IL, USA). Protein content was measured using a standard BCA protein assay.

### Macrophage activation

Bone marrow derived macrophages were plated in triplicate at a density of 0.5 × 10^6^ cells/ml. C5a was added in a concentration range of 0.2, 2, 20 or 200 nM and incubated overnight at 37°C. To investigate the effect of C5a on cytokine release, IL-6 and MCP-1 were measured by means of ELISA (BD Bioscience, San Diego, CA, USA). Cells were lysed for RNA isolation.

### RNA isolation, cDNA synthesis and qPCR

Guanidine thiocyanate was used to extract total RNA from BMDMs 27. RNA was reverse transcribed by M-MuLV reverse transcriptase (RevertAid, MBI Fermentas, Leon-Roth) and used for quantitative analysis of mouse genes (Table S1) with an ABI PRISM 7700 Taqman apparatus (Applied Biosystems, Foster City, CA, USA). Murine hypoxanthine phosphoribosyltransferase and murine ribosomal protein 27 (RPL27) were used as standard housekeeping genes.

### Annexin V staining

Annexin V staining was performed to detect apoptosis. vSMC and H5V were plated in quadruplicate at 0.4 × 10^6^ cells/ml in medium. After attachment of the cells, C5a was added overnight in a concentration range of 2, 20 or 200 nM, which is based on previous literature 28 or H_2_O_2_ as a positive control. Cells were washed and stained with fluorochrome-conjugated Annexin V. To distinguish late stage apoptosis from early apoptosis, cells were subsequently stained with Propidium Iodide Staining Solution. In late stage apoptosis the cell membrane loses its integrity, these cells then become double-positive for Annexin V and Propidium Iodide. Analysis was performed by flow cytometry (FACS CantoII, BD Bioscience, Breda, The Netherlands).

### Statistical analysis

Data are expressed as mean ± SEM. A Fisher's exact test was used to measure the statistical significance between the amounts of plaque dissections. A two-tailed Student's *t*-test was used to compare individual groups. Multiple group comparisons were analysed by two-way anova. Non-parametric data were analysed using a Mann–Whitney *U*-test. A value of *P* < 0.05 was considered significant.

## Results

### Lesion size, C5a and C5aR expression

**Figure 1:**
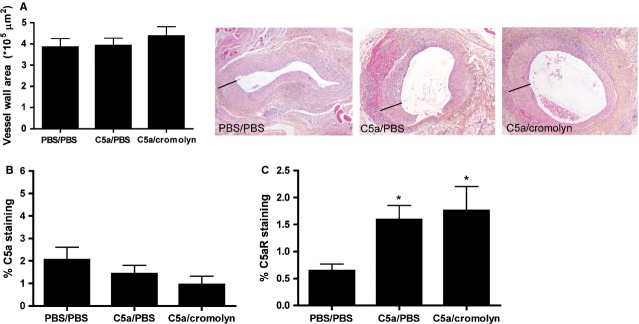
Lesion size, C5a and C5a Receptor expression. Local application of C5a in late stage atherosclerosis did not result in a change in lesion size (**A**). Three representative pictures (40× magnifications) show haematoxylin-phloxine saffron (HPS) stained vessel wall segments of all treatment groups, used for measurement of lesion size and plaque rupture analysis. Lines indicate vessel wall thickness. Immunohistochemical staining revealed no changes in C5a expression between treatment groups (**B**). However, C5a receptor (C5aR) expression was significantly up-regulated in mice treated with C5a (**C**) (PBS/PBS treated mice: *n* = 8; C5a/PBS treated mice: *n* = 9; C5a/cromolyn treated mice: *n* = 7; *P < 0.05 compared to PBS/PBS).

To investigate the effect of C5a on the composition of advanced plaques, age-and weight-matched apoE^−/−^ mice with advanced lesions were treated locally with C5a or PBS. No differences were found in plasma cholesterol levels; also, plasma IL-6 concentration did not differ between groups (Fig. S1). Analysis of atherosclerotic plaques revealed no difference between groups in lesion size, measured as vessel wall area (PBS: 3.9 ± 0.4 × 10^5^ μm^2^; C5a: 3.9 ± 0.3 × 10^5^ μm^2^; C5a/cromolyn: 4.4 ± 0.4 × 10^5^ μm^2^; Fig.1A).

The presence of C5a and C5aR in the atherosclerotic lesions was determined by immunohistochemical stainings. No differences in C5a expression were found between the three groups (PBS: 2.1% ± 0.6%; C5a: 1.4% ± 0.4% *P* = 0.4 compared to PBS; C5a/cromolyn: 1.0% ± 0.4% *P* = 0.2 compared to PBS; Fig.1B). Interestingly, treatment with C5a resulted in a significant increase in C5aR expression in the lesions (PBS: 0.6% ± 0.1%; C5a: 1.6% ± 0.3% *P* < 0.01 compared to PBS; C5a/cromolyn: 1.8% ± 0.4% *P* < 0.05 compared to PBS; Fig.1C). Both C5a and C5a receptor staining was distributed heterogeneously within the lesions, suggesting expression by multiple cell types in the intimal layer. Consistent with literature 7,8, we found C5a and C5aR to be expressed by macrophages, endothelial cells and vascular smooth muscle cells (Fig. S2).

### Plaque morphology

**Figure 2:**
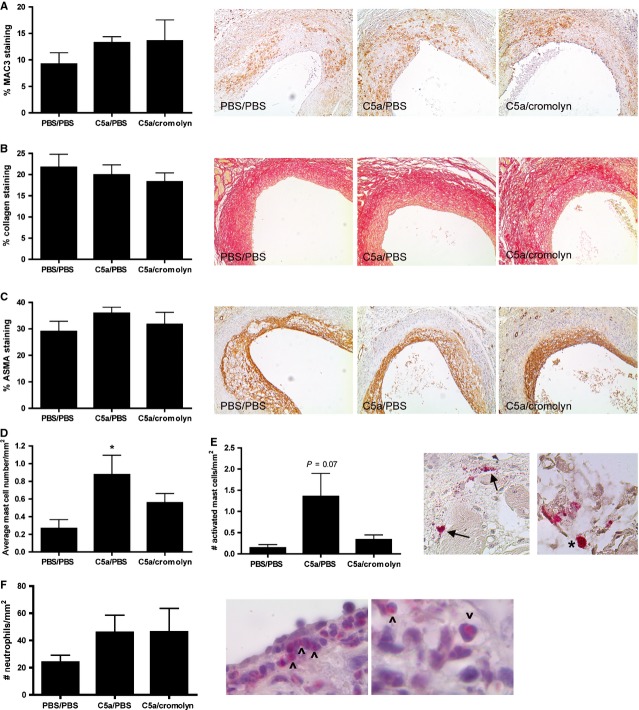
Plaque morphology after local treatment with C5a. Plaque composition was investigated by staining for macrophages (MAC3, **A**), collagen (Sirius Red, **B**) and smooth muscle cells (ASMA,**C**). Representative pictures (100× magnifications) show immunohistochemical staining of all treatment groups. Also, the total amount of mast cells in the perivascular tissue was determined (**D**) as well as the amount of activated mast cells (**E**). Pictures show activated mast cells (arrows) with granules present in the vicinity of the cell. Also, a resting mast cell (*) is shown with all granules contained inside the cell. Neutrophils in the intima were stained with a naphtol CAE staining (**F**). Pictures show neutrophils stained light pink with clearly visible segmented nuclei (^) (PBS/PBS treated mice: *n* = 8; C5a/PBS treated mice: *n* = 9; C5a/cromolyn treated mice: *n* = 7; *P < 0.05 compared to PBS/PBS).

Macrophage, smooth muscle cell and collagen content were measured to establish plaque phenotype. C5a treatment did not affect macrophage content as measured by MAC3 staining (PBS: 13.2% ± 1.2%; C5a: 13.3% ± 3.9%; C5a/cromolyn: 9.2% ± 2.2%; Fig.2A). Also, the collagen content (PBS: 21.7% ± 3.1%; C5a: 19.9% ± 2.4%; C5a/cromolyn: 18.3% ± 2.1%; Fig.2B) and the amount of smooth muscle cells (PBS: 29.0% ± 3.9%; C5a: 36.9% ± 2.3%; C5a/cromolyn: 31.7% ± 4.6%; Fig.2C) did not differ between groups.

Local application of C5a resulted in a trend towards an increased amount of activated mast cells (*P* = 0.07; Fig.2E), which was decreased in mice treated with cromolyn. Moreover, the total amount of perivascular mast cells was significantly increased after C5a treatment, which could partly be inhibited by cromolyn (PBS: 0.3 ± 0.1 mast cells/mm^2^; C5a: 0.9 ± 0.2 mast cells/mm^2^; C5a/cromolyn: 0.6 ± 0.1 mast cells/mm^2^; Fig.2D). These findings are consistent with our previous data, where we established that local treatment with a specific mast cell activator results in activation of perivascular mast cells and subsequent recruitment of newly infiltrating mast cells into the vessel wall 19.

Neutrophil count in the intima did not differ between the three treatment groups (Fig.2F). Also, no signs of leaky vessels were detected, defined as the presence of erythrocytes outside vascular-like structures in the intima; excluding areas in which erythrocytes were present because of plaque disruptions 18.

### Plaque disruptions and fibrin depositions in C5a treated groups

**Figure 3:**
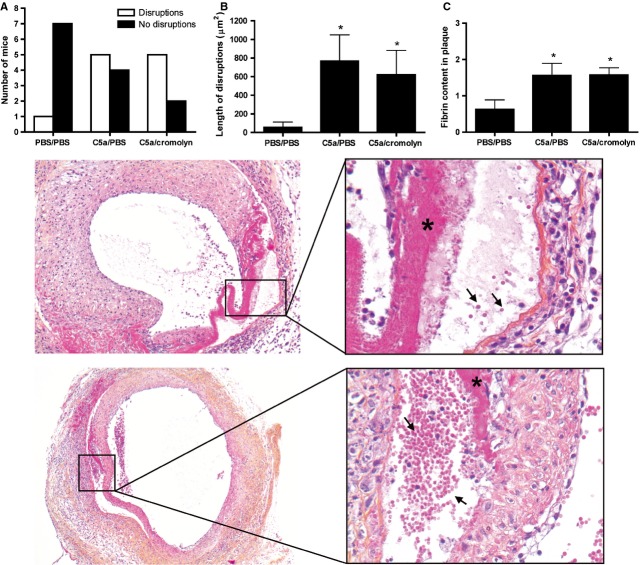
Increased amount of plaque disruptions and fibrin depositions after treatment with C5a. Three days after local application of C5a in late stage atherosclerosis, increased amount of plaque disruptions, as defined by the presence of erythrocytes in the lesions (arrows) with concomitant fibrin depositions, were present in the treated groups (**A**), while also the length of the disruptions was increased (**B**). Fibrin content (*) was significantly increased in mice treated with C5a (**B**). Cromolyn, a common mast cell stabilizer, did not reduce this effect (PBS/PBS treated mice: *n* = 8; C5a/PBS treated mice: *n* = 9; C5a/cromolyn treated mice: *n* = 7; *P < 0.05 compared to PBS/PBS).

Haematoxylin-phloxine saffron staining was used for further analysis of lesion morphology. Interestingly, local treatment with C5a showed an increase in the amount of plaque disruptions from 13% in the PBS treated group up to 56% in the C5a treated group. Plaque disruptions were defined as the presence of erythrocytes in the lesions with concomitant fibrin depositions. Moreover, additional analysis revealed a significant increase in the length of disruptions after C5a application (*P* < 0.05). Cromolyn treatment did not reduce the number (71%) or length of the disruptions (Fig.3A and B). Analysis of fibrin content in the plaque revealed a significant increase after treatment with C5a (Fig.3C; *P* < 0.05). Again, cromolyn application did not reduce this effect, indicating a mast cell independent role of C5a in plaque rupture. To explain these findings, we next performed *in vitro* studies to evaluate the direct effect of C5a on macrophages, smooth muscle cells and endothelial cells.

### *In vitro* activation by C5a

**Figure 4:**
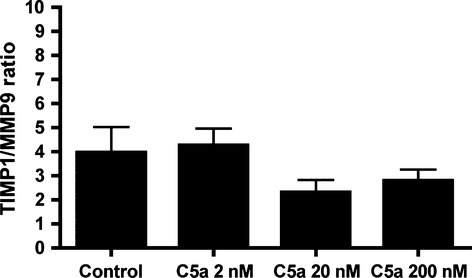
TIMP1/MMP9 ratio after *in vitro* stimulation of BMDMs with C5a. Expression levels of TIMP1/MMP9 ratio in BM derived macrophages showed no significant differences after activation with increasing concentrations of C5a.

Activation of BMDMs by C5a resulted in an increase in MCP-1 secretion of almost seven fold (200 nM C5a: 888.0 pg/ml; control: 128.8 pg/ml; *P* < 0.001). No changes were detected in IL-6 secretion by C5a activated macrophages. Collagen synthesis rate of smooth muscle cells remained unchanged after treatment with C5a (data not shown). Furthermore, the TIMP1/MMP9 ratio, as a marker of the proteolytic status of C5a stimulated macrophages, did not significantly change after C5a stimulation (Fig.4).

Treatment of endothelial cells with a concentration range of C5a (2–200 nM) did not induce changes in the expression of the adhesion molecules V-CAM, I-CAM or PECAM (Fig. S3).

### *In vitro* apoptosis

**Figure 5:**
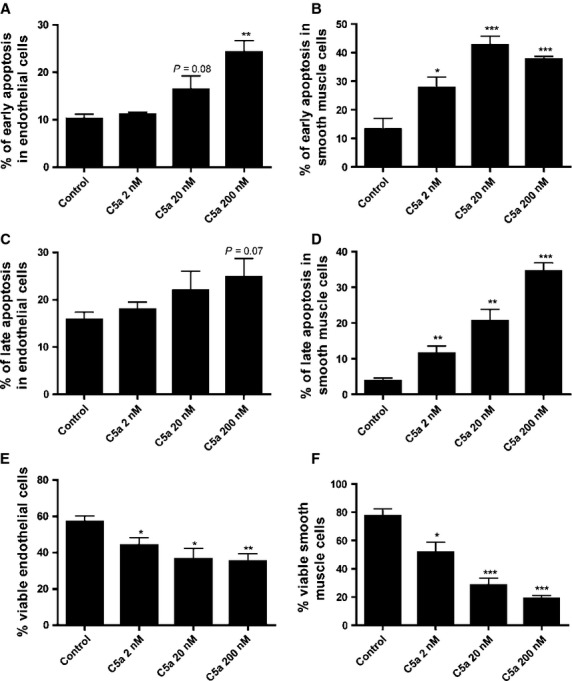
Increased amount of early and late apoptosis of smooth muscle cells and endothelial cells after treatment with C5a *in vitro*. Treatment of smooth muscle cells *in vitro* by C5a 2 nM, C5a 20 nM and C5a 200 nM showed a marked dose-dependent increase in both early (**B**) and late apoptosis (**D**). Consequently, the amount of viable smooth muscle cells after C5a stimulation was significantly decreased (**F**). Also, early apoptosis (**A**) and late apoptosis (**C**) of endothelial cells were increased after C5a stimulation. The amount of viable endothelial cells showed a dose-dependent decrease (**E**). Both endothelial cell and smooth muscle cell apoptosis can contribute to plaque destabilization (*P < 0.05; **P < 0.01; ***P < 0.001).

One of the features of plaque instability is smooth muscle cell apoptosis 3,4. To investigate the effect of C5a on smooth muscle cell viability, we added C5a in concentrations ranging from 2, 20 to 200 nM to cultured smooth muscle cells. Interestingly, we found a clear dose-dependent increase in smooth muscle cell apoptosis after exposure to C5a. Both the percentage of early apoptosis (200 nM C5a: 37.8% ± 0.9%; control: 13.2% ± 3.9%; *P* < 0.001; Fig.5B), as the percentage of late stage apoptosis (200 nM C5a: 34.6% ± 2.3%; control: 3.8% ± 0.8%; *P* < 0.0001; Fig.5D), were significantly increased compared to the negative control. Consequently, the amount of viable cells was drastically decreased after C5a treatment (200 nM C5a: 20.0% ± 2.2%; control: 77.4% ± 5.2%; *P* < 0.0001; Fig.5F).

Since disturbed endothelial lining along the luminal site of the vessel pre-disposes to plaque erosions and instability as well, we stimulated endothelial cells with the same C5a concentration range. Early apoptosis was significantly increased compared to the negative control (200 nM C5a: 24.3% ± 2.4%; control: 10.3% ± 0.9%; *P* < 0.005; Fig.5A) in a dose-dependent manner. Also, a concomitant trend towards increased late stage apoptosis was detected (200 nM C5a: 24.9% ± 3.9%; control: 15.9% ± 1.6%; *P* = 0.07; Fig.5C). The percentage of viable cells was significantly decreased after treatment with C5a (200 nM C5a: 35.3% ± 4.1%; control: 57.2% ± 3.1%; *P* < 0.01; Fig.5E). Cromolyn did not affect C5a-induced apoptosis of either endothelial cells or smooth muscle cells (data not shown).

As it was previously established that C5a can directly induce cellular apoptosis *via* C5aR, resulting in activation of the caspase cascade 29–31, we aimed to investigate whether C5a can also directly induce apoptosis by up-regulation of caspases in endothelial cells. We measured mRNA levels of caspase-1, caspase-3 and Bax after stimulation with 2, 20 or 200 nM C5a. We did not detect any changes between caspase-1 or Bax expression, however, we observed a significant dose-dependent increase in caspase-3 expression after C5a stimulation (Fig. S4). No changes were detected in the pro-apoptotic cytokine TNF-α, suggesting that C5a induced apoptosis is not mediated *via* local production of TNF-α by the endothelial cells.

### *In vivo* apoptosis

**Figure 6:**
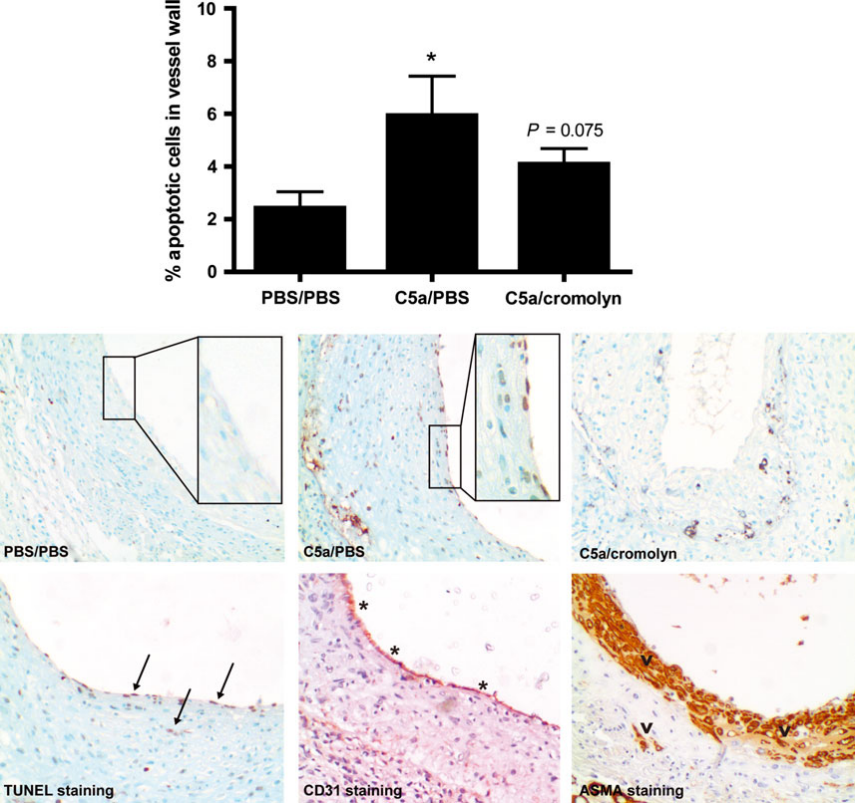
Increased apoptosis in the vessel wall. Apoptosis measurement is depicted as the percentage of apoptotic cells of the total number of cells within the vessel wall. Perivascular treatment with C5a resulted in an increase in apoptosis (*P < 0.05), which was not affected by cromolyn treatment. Top pictures show representative TUNEL stained sections of all three treatment groups (200× magnifications). Enlargements demonstrate endothelial cell apoptosis in C5a treated groups, which is mostly absent in control mice (400× magnifications). Pictures in the lower panel show representative micrographs of consecutive slides (200× magnifications). From left to right are shown: TUNEL positive staining for apoptotic cells (arrows); CD31 positive staining for endothelial cells (*) and alpha smooth muscle cell actin positive staining (^) (PBS/PBS treated mice: *n* = 8; C5a/PBS treated mice: *n* = 9; C5a/cromolyn treated mice: *n* = 7; *P < 0.05 compared to PBS/PBS).

To confirm our *in vitro* results, we performed a TUNEL staining to detect apoptosis within the atherosclerotic plaque. Local application of C5a resulted in a significant increase in the percentage of apoptotic cells in the vessel wall, thereby validating our *in vitro* findings (PBS: 2.4% ± 0.6% of the total cell population; C5a: 5.9% ± 1.4%; *P* < 0.05; Fig.6). Cromolyn treatment slightly reduced this effect, however there was still an increase in apoptotic cells apparent (PBS: 2.4% ± 0.6%; C5a/cromolyn: 4.1% ± 0.6%; *P* = 0.075 compared to PBS; Fig.6). Apoptotic cells were apparent along the endothelial lining of luminal site of the vessel, as well as in smooth muscle cell rich areas. These data identify the induction of apoptosis as a potential mechanism *via* which C5a can induce plaque disruptions.

## Discussion

This study describes a causal role for complement factor C5a in acute atherosclerotic plaque disruptions, which was studied by the perivascular application of C5a in advanced atherosclerotic lesions. Acute activation by C5a resulted in increased amount of plaque disruptions with concomitant intraplaque haemorrhage and fibrin depositions. *In vitro* stimulation with C5a led to a striking increase in smooth muscle cell and endothelial cell apoptosis. Moreover, increased apoptosis was confirmed in the vessel wall after C5a treatment, which may contribute to the plaque disruptions observed in this study.

Previously, we have demonstrated that local application of C5a at time of graft placement aggravates vein graft disease *via* activation of perivascular mast cells 14. In that previous study, mice received C5a treatment only during the first days after surgery; therefore C5a had the most prominent effects on the initiation of the lesion. In this initial process both attraction and activation of mast cells have shown to be important. Interestingly, in this study, where effects of C5a on advanced lesions were investigated, results are indicative of a mast cell independent mechanism as the mast cell stabilizer cromolyn did not prevent the C5a induced effects on plaque disruptions. These effects can be explained by differences in relative cellular content of early *versus* advanced lesions. In early lesions recruitment of inflammatory cells, possibly *via* mast cell derived chemokines such as MCP-1, plays a prominent role, while in advanced lesions a loss of matrix deposition and increased apoptosis are detrimental for plaque stability. The mechanism behind C5a activation may therefore differ between early as compared to advanced stages of atherosclerosis. Indeed, in our previous study, we have established that C5a attracts and activates mast cells, resulting in a more profound influx of inflammatory cells and accelerated vein graft disease. In mature lesions however, the amount of immune cells present in the vessel wall is already strongly increased. Although, we again observe an increased number of mast cells in this study, and mast cell proteases have been implicated in plaque stability and apoptosis 19,32,33, C5a itself may have more direct and pronounced effects on other cell types involved in maintaining plaque stability, such as the smooth muscle cell.

Features of an unstable atherosclerotic plaque, prone to rupture, have been described extensively 34. A thin fibrous cap devoid of collagen and smooth muscle cells, a large a-cellular necrotic core and increased inflammation and neovascularization are detrimental for plaque stability. Disruptions described in the current experiment are not equal to plaque ruptures in the classical definition, in which a thin cap covering a lipid core ruptures, resulting in atherothrombosis 35. This may be because of the fact that the vein grafts described in this study do not contain large necrotic cores similar to human plaques, but smaller necrotic cores distributed heterogeneous throughout the intima. However, we emphasize that, as of yet, there have not been any mouse models described that completely resemble human plaque rupture, and its complex lesion morphology 36–38. In contrast to many other mouse models, this vein graft model does exhibit a complex morphology including neovascularization, calcifications and disruptions consisting of fibrin depositions and intraplaque haemorrhage, which has extensively been described by De Vries *et al*. 18. We therefore believe that this model can make a valuable contribution to research regarding the underlying mechanisms behind plaque disruptions.

To elucidate the mechanism by which C5a causes plaque disruptions, we investigated the effect of C5a *in vitro* on various cell types. Speidl *et al*. have previously demonstrated an increase in MMP expression after C5a administration to human macrophages 39. Because collagen turnover is important for the integrity of the fibrous cap, we determined the ratio of TIMP1/MMP9 expression after treating murine macrophages with C5a. Consistent with our *in vivo* data, where we did not observe any effects on lesional collagen content, we did not observe any changes in the TIMP1/MMP9 ratio, while we also did not observe any effects on smooth muscle cell dependent collagen production. Taken together, these data suggest that C5a does not acutely affect plaque stability *via* interference in matrix homoeostasis.

Next, we investigated the effect of C5a on cellular apoptosis, a pathway which has also been suggested to contribute to plaque instability 40,41. Intriguingly, a marked dose-dependent increase in both early and late apoptosis was detected when administering C5a to smooth muscle cells as well as to endothelial cells. These results are consistent with previous findings that C5a can induce apoptosis of adrenomedullary cells and neuronal cells 30,31. We here demonstrate that C5a causes a striking increase in apoptosis of smooth muscle cells. In late stage atherosclerosis, smooth muscle cell apoptosis is responsible for a potent inflammatory response, loss of collagen and thinning of the fibrous cap, whereas it causes no inflammation or thrombosis in normal arteries 42. Specific apoptosis of smooth muscle cells in established atherosclerotic lesions resulted in plaques with unstable features 43. Also, it has been shown that induction of smooth muscle cell apoptosis by specific SIRT1 deletion results in reduced cap/intima ratio 44. Together, these data highlight the importance of viable smooth muscle cells to the fibrous cap. Furthermore, we detected a remarkable increase in endothelial cell apoptosis after addition of C5a. Previously, it has been described that C5a induces apoptosis of endothelial cells 29. Induction of endothelial cell apoptosis may result in erosions at the luminal site of atherosclerotic lesions, making the surface prone to thrombus formation and subsequent acute cardiovascular syndromes 45. Indeed, local C5a treatment resulted in an increased percentage of apoptotic cells within the atherosclerotic plaque. However, it should be noted that most of the cells in the lesion are C5aR positive; therefore we cannot completely exclude the possibility of effects of C5a on other C5aR positive cells besides endothelial and smooth muscle cells.

Although the local concentration of C5a *in vivo* was artificially created in this study, it should be realized that previous reports described an increase in serum C5a in patients suffering from cardiovascular disease, furthermore, C5a levels did correlate with cardiovascular events and late lumen loss in drug eluting stents 17,46. C5a in the plaque and adventitia may thus derive *via* diffusion through peri-vessels; also, it may be locally derived from various cell types in the vessel wall, such as smooth muscle cells, macrophages and endothelial cells.

In conclusion, we here demonstrate a causal role for C5a in the induction of atherosclerotic plaque disruptions. C5a exposure resulted in a marked increase in endothelial cell and smooth muscle cell apoptosis *in vitro*. Moreover, increased apoptosis *in vivo* was observed, which may contribute to plaque destabilization. With respect to rheumatoid arthritis and psoriasis, phase Ib/IIa clinical trials for a C5a inhibitor have already been completed 47, and a similar therapeutic strategy using complement factor C5a as a target may be promising in the prevention of acute cardiovascular events.

## Supporting Information

**Figure S1** Plasma IL-6 concentration did not differ between the three treatment groups. (PBS/PBS treated mice: *n* = 8; C5a/PBS treated mice: *n* = 9; C5a/cromolyn treated mice: *n* = 7). **Figure S2** Figure 2. Q-PCR analysis of mRNA, isolated from smooth muscle cells, macrophages and endothelial cells, show the expression of both HC (C5a) and C5aR (A). **Figure S3** Mouse endothelial cells (H5V) were stimulated with increasing concentrations of C5a *in vitro*. **Figure S4** Mouse endothelial cells (H5V) were stimulated with 2, 20, or 200 nm C5a *in vitro*. **Table S1** QPCR primers used for the *in vitro* experiments.
