# Supplementary material for: Complement factor C5a induces atherosclerotic plaque disruptions
Source: J Cell Mol Med. 2014 Aug 15;18(10):2020–30. doi: 10.1111/jcmm.12357 (PMC4244017; doi:10.1111/jcmm.12357)

**Complement factor C5a induces atherosclerotic plaque disruptions**

**Anouk Wezel^1,2^, Margreet R. de Vries^2,3^, H. Maxime Lagraauw^1^, Amanda C. Foks^1^, Johan Kuiper^1^, Paul H.A. Quax^2,3^, *Ilze Bot^1,2^**

*Short title: Wezel et al. C5a induces plaque disruptions*

^1^Division of Biopharmaceutics, Leiden Academic Centre for Drug Research, Leiden University, Leiden, The Netherlands, ^2^Department of Surgery, and ^3^Einthoven Laboratory for Experimental Vascular Medicine, Leiden University Medical Center, Leiden, The Netherlands

# Corresponding authors:

Ilze Bot, Division of Biopharmaceutics, Leiden Academic Centre for Drug Research, Gorlaeus Laboratories, Leiden University, Einsteinweg 55, 2333 CC, Leiden, The Netherlands. Paul Quax, ^2^Department of Surgery Leiden, University Medical Center, Leiden, The Netherlands

# Tel.nr: + 31 (0)715276213; +31 (0)715261584

# e-mail: [**i.bot@lacdr.leidenuniv.nl**](mailto:i.bot@lacdr.leidenuniv.nl)**;** [**p.h.a.quax@lumc.nl**](https://mail.lumc.nl/owa/redir.aspx?C=s4w1WTqPfEW2JycH0MamfoHIpXP5PtAITJKoUCzZ1_F2zz6fTsgOdGHE9w0URMTkHsQrYLk703w.&URL=mailto%3Ap.h.a.quax@lumc.nl)

**Supplemental Figure Legends**

**Supplemental Table 1. QPCR** primers used for the *in vitro* experiments.

| **Gene** | **Forward primer** | **Reversed primer** |
| --- | --- | --- |
| **TIMP1** | ACACCCCAGTCATGGAAAGC | CTTAGGCGGCCCGTGAT |
| **MMP9** | CCCTGGAACTCACACGACATCTTC | CTCATTTTGGAAACTCACACGCCAG |
| **I-CAM-1** | GTCCGCTTCCGCTACCATCAC | GGTCCTTGCCTACTTGCTGCC |
| **V-CAM-1** | AGACTGAAGTTGGCTCACAATTAAGAAG | AGTAGAGTGCAAGGAGTTCGGG |
| **PECAM-1** | GTCTTGTCGCAGTATCAGAATTTCAG | TACCAGGCCGCTTCTCTTGA |
| **HC (C5a)** | ACACTGCGACTCTTCTGGTCACT | CCAGGTTGGCATTGGTACAGCTC |
| **C5aR** | GACCCCATAGATAACAGCA | CAGAGGCAACACAAAACCCA |
| **Caspase-1** | GGCATTAAGAAGGCCCATATAGAGA | TGAGCCCCTGACAGGATGTC |
| **Caspase-3** | AACTTCCATAAGAGCACTGGAATGTC | ACTTGGTATTTCAGGCCCATGA |
| **Bax** | CGTGGTTGCCCTCTTCTACTTT | TGATCAGCTCGGGCACTTTA |
| **TNFα** | GCCTCTTCTCATTCCTGCTTGTG | ATGATCTGAGTGTGAGGGTCTGG |
| **HPRT** | TTGCTCGAGATGTCATGAAGGA | AGCAGGTCAGCAAAGAACTTATAG |
| **RPL27** | TGAAAGGTTAGCGGAAGTGC | TTTCATGAACTTGCCCATCTC |

**Supplemental Figure 1.** Plasma IL-6 concentration did not differ between the three treatment groups. (PBS/PBS treated mice: n=8; C5a/PBS treated mice: n=9; C5a/cromolyn treated mice: n=7).


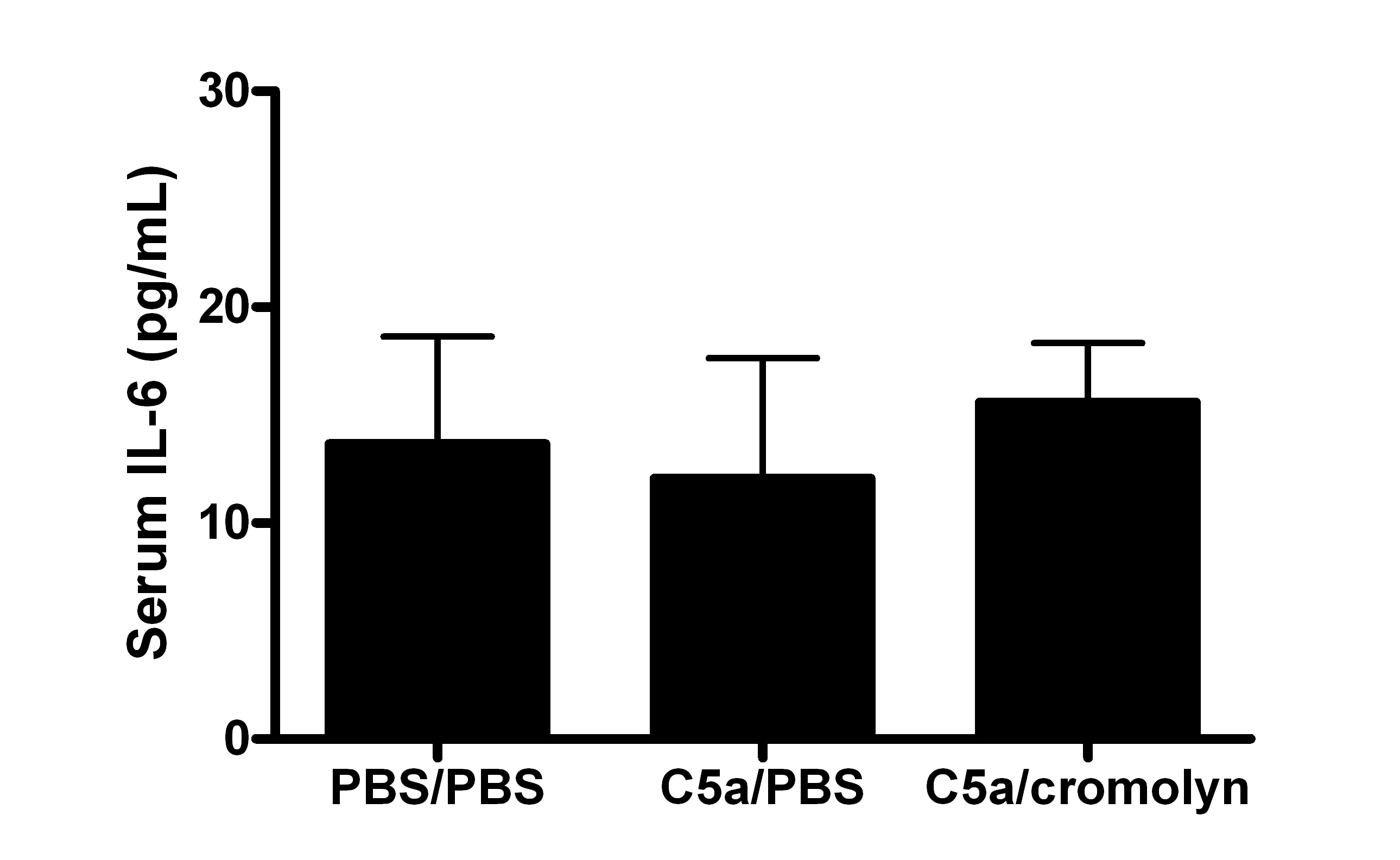


**Supplemental Figure 2.** Q-PCR analysis of mRNA, isolated from smooth muscle cells, macrophages and endothelial cells, show the expression of both HC (C5a) and C5aR (A). (B) Representative micrographs (C5a(*), C5aR(^); CD31: 200x magnification. MAC-3, ASMA: 100x magnification) indicate that cells expressing C5a and C5aR in the vessel wall are mostly smooth muscle cells, endothelial cells and macrophages.


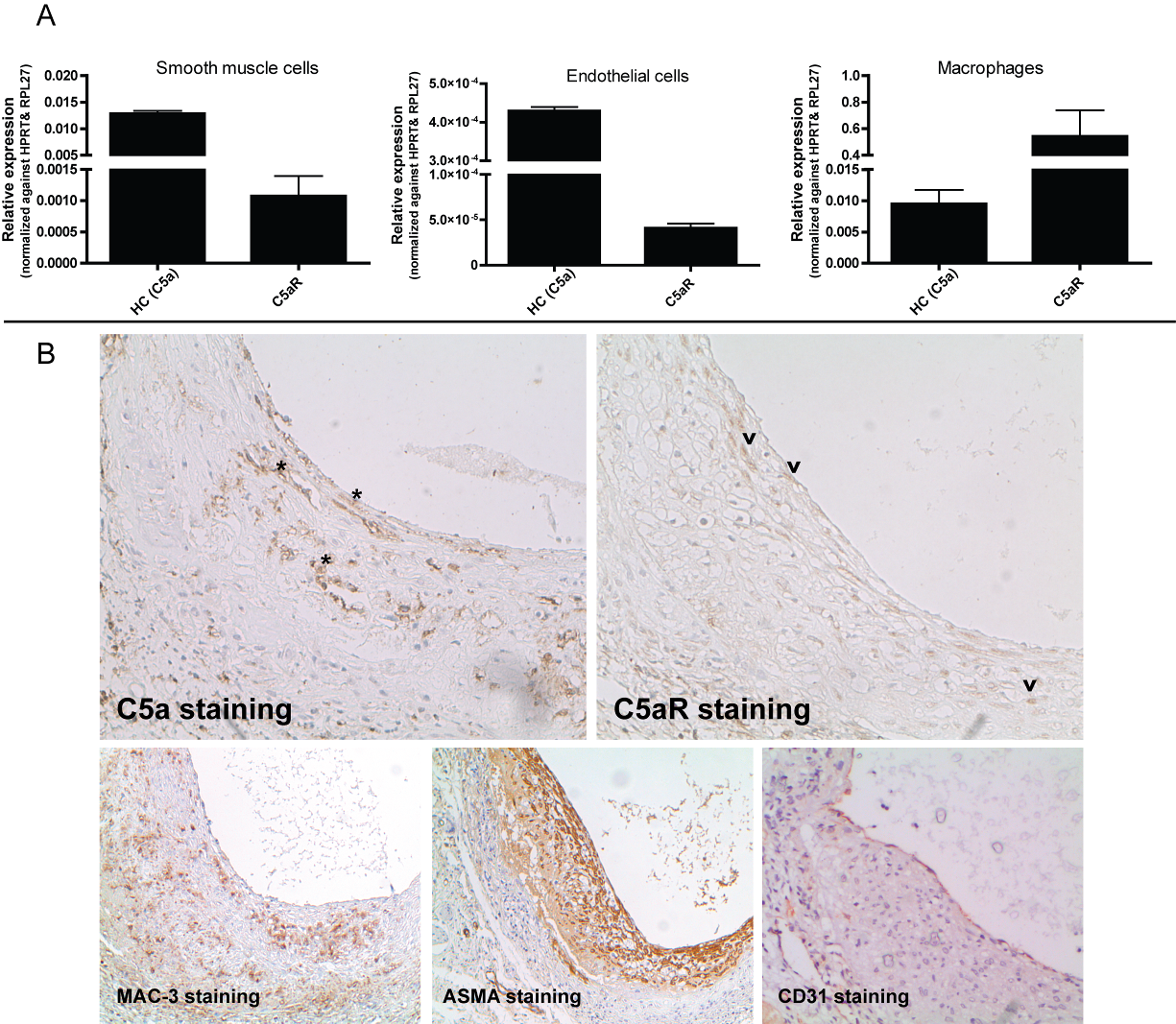


**Supplemental Figure 3.** Mouse endothelial cells (H5V) were stimulated with increasing concentrations of C5a *in vitro.* TNFα stimulation was used as a positive control and resulted in a significant upregulation of both V-CAM and I-CAM. Stimulation with C5a however did not result in a changed expression of V-CAM, I-CAM or PECAM. (*P<0.05; *** P<0.001).

**
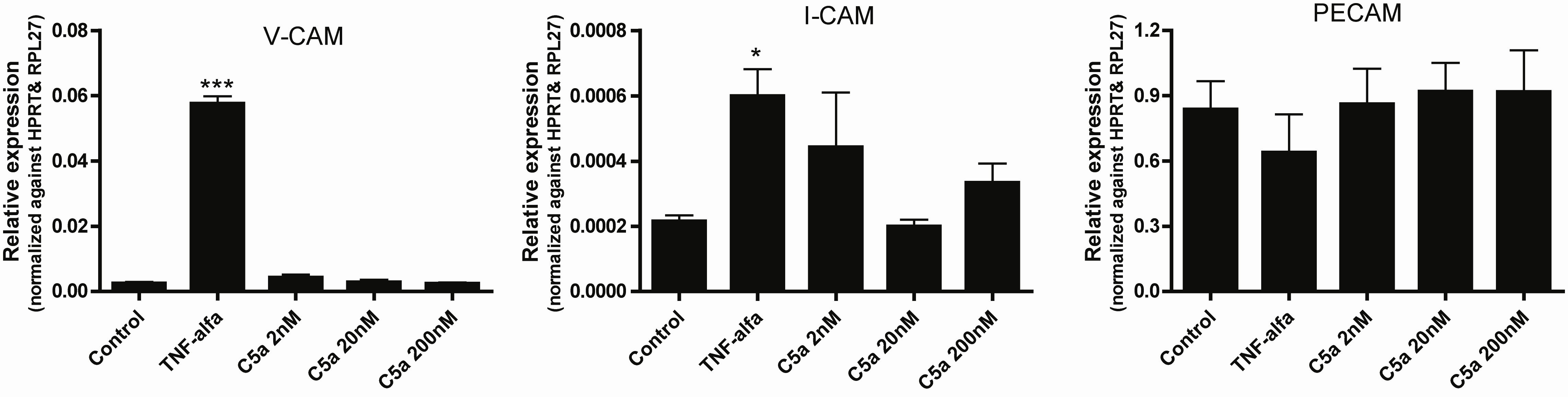
**

**Supplemental Figure 4.** Mouse endothelial cells (H5V) were stimulated with 2 nm, 20 nm, or 200 nm C5a *in vitro*. No changes were observed in the gene expression levels of caspase-1 or Bax, however the expression of caspase-3 was significantly and dose-dependently increased after C5a treatment. The expression level of the pro-apoptotic cytokine TNFα did not show any differences after C5a stimulation. (*P<0.05; ** P<0.01).


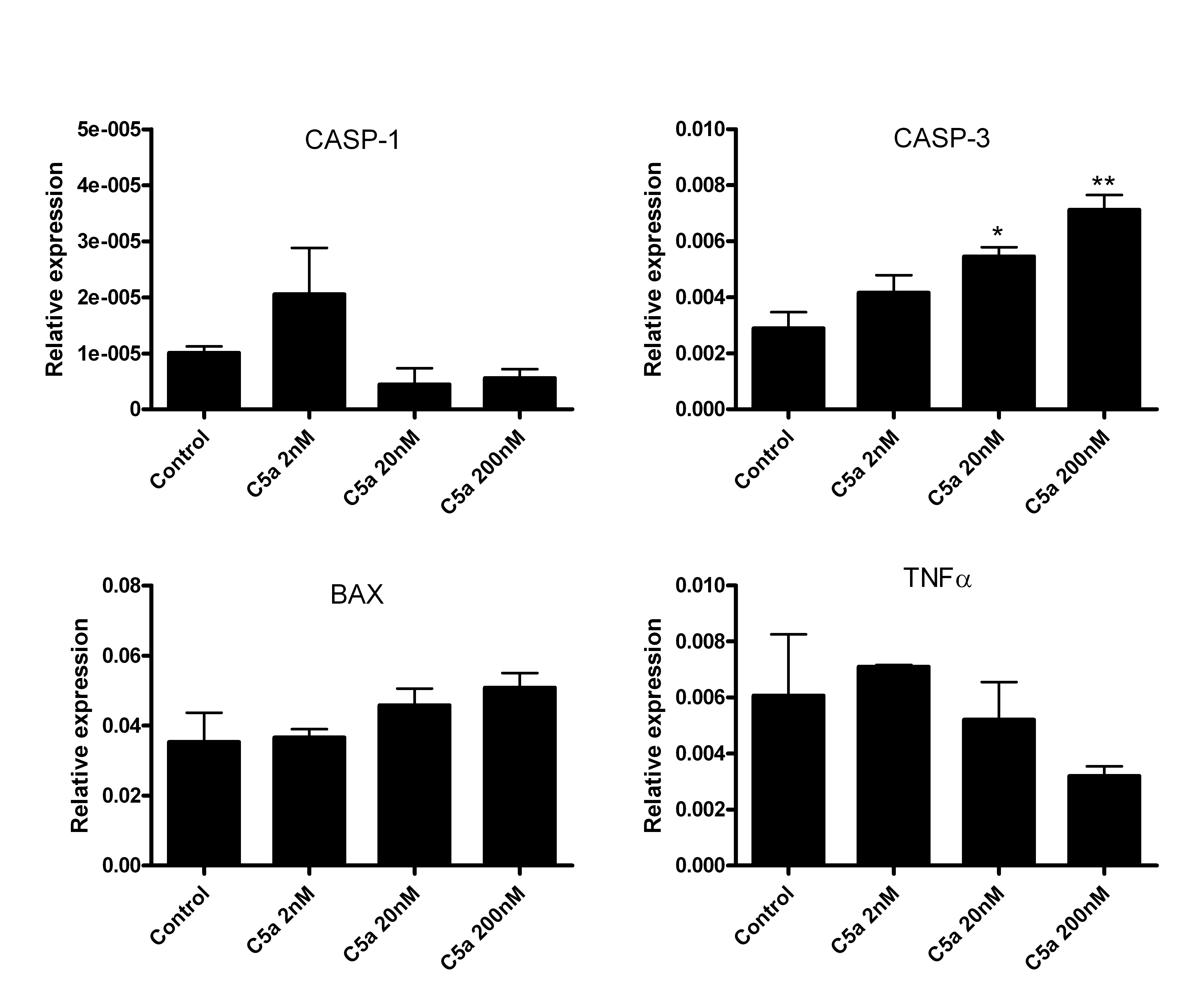

Supplement: Supplementary file 1 [file jcmm0018-2020-sd1.doc]
